# Eribulin Provides a Remarkable Effect in Trabectedin-Resistant Myxoid Liposarcoma

**DOI:** 10.1155/2020/8873185

**Published:** 2020-11-12

**Authors:** Hironari Tamiya, Hideaki Sabe, Katsunari Yamashita, Yoshinori Imura, Toru Wakamatsu, Satoshi Takenaka

**Affiliations:** ^1^Department of Orthopedic Surgery, Osaka International Cancer Institute, Osaka 541-8567, Japan; ^2^Department of Rehabilitation, Osaka International Cancer Institute, Osaka 541-8567, Japan

## Abstract

Adriamycin-based chemotherapy is commonly used for malignant soft tissue sarcoma including myxoid liposarcoma. However, in the case of unavailability or failure of the adriamycin-based regimen, trabectedin or eribulin can produce a good antitumor effect for myxoid liposarcoma. We relate the experience of a 64-year-old female with myxoid liposarcoma, who noticed a nodule on her left thigh and visited our institute. At initial presentation, the tumor was 18.7 cm in diameter, and the magnetic resonance imaging (MRI) showed a malignant lipomatous tumor with a myxoid component. We recommended that she undergo treatment; however, she refused. Three years later, the tumor had grown larger, so she finally decided to undergo treatment. A needle biopsy revealed a myxoid liposarcoma. The tumor massively involved the neurovascular structures; we thus determined that hip disarticulation was inevitable. Two years later, metastases in the right thigh, left lung, right ileum, and abdominal space were pointed out and chemotherapy was initiated. Adriamycin was unusable due to cardiac dysfunction, so trabectedin was administered; however, the tumors progressed. Eribulin was subsequently started and has been considerably effective for more than 2 years without severe adverse effects. In conclusion, we experienced a case showing the remarkable and long-lasting effect of eribulin against trabectedin-resistant myxoid liposarcoma.

## 1. Introduction

Liposarcoma is a relatively common malignant soft tissue tumor. Histological subtype classification is important because clinical features, including sensitivity to chemotherapy and radiotherapy and overall survival in each subtype, vary. Liposarcoma is categorized into well-differentiated, dedifferentiated, pleomorphic, and myxoid liposarcoma. Well-differentiated liposarcoma provides a good prognosis with a 5-year survival rate of about 100% [1]. In contrast, pleomorphic and dedifferentiated liposarcomas have a high-grade malignancy, in which 5-year overall survival rates are reported to be around 60% and 70%, respectively [2, 3]. Myxoid liposarcoma is another subtype of high-grade potential, and overall survival varies depending on the presence of round-cell components [4].

As a treatment, surgical resection is the mainstay, which is similar to other types of sarcoma. Well-differentiated liposarcoma is low grade, in which chemotherapy or radiotherapy is not effective and surgery is the only option, unless it differentiates into the dedifferentiated type. Pleomorphic and dedifferentiated liposarcomas also respond poorly to chemotherapy and radiotherapy; therefore, surgical resection with a negative margin is preferred [1]. On the other hand, chemotherapy and radiotherapy provide a good response in myxoid liposarcoma [5]. As a chemotherapeutic treatment, the adriamycin-based regimen is standard for malignant soft tissue sarcoma [6]. However, cardiotoxicity or hematotoxicity is occasionally problematic [7].

Three anticancer agents for soft tissue sarcoma have recently been developed. In the 2010s, pazopanib, trabectedin, and eribulin were used for soft tissue sarcoma and the effectiveness of each was reported for a certain type of the sarcoma.

In a previous report, eribulin was found to be superior to dacarbazine in the dedifferentiated and pleomorphic types, but not in myxoid liposarcoma [8]. There is still no evidence of how effective eribulin is for myxoid liposarcoma.

## 2. Case Presentation

**Figure 1:**
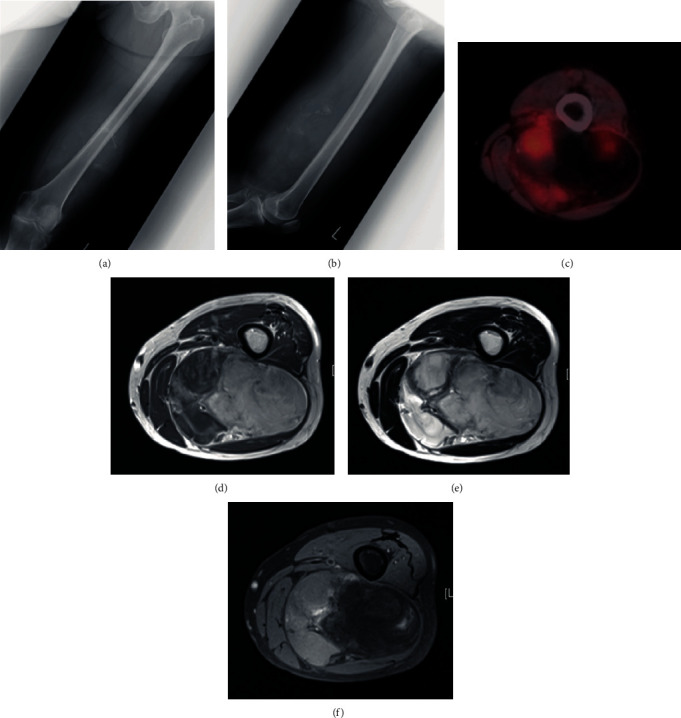
Images of myxoid liposarcoma in the left thigh at initial visit. Plain radiograph in (a) anteroposterior and (b) lateral view, (c) PET, (d) T1-weighted, (e) T2-weighted, and (f) T1-weighted fat suppressed MRI.

**Figure 2:**
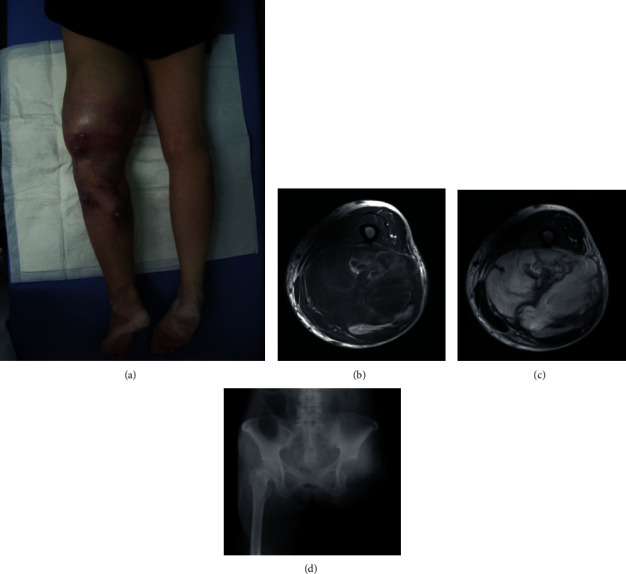
Tumor at the moment of revisiting and hip disarticulation for tumor excision. Tumor in (a) macroscopic image, (b) T1-weighted, and (c) T2-weighted MRI. (d) Plain radiograph after left hip disarticulation.

**Figure 3:**
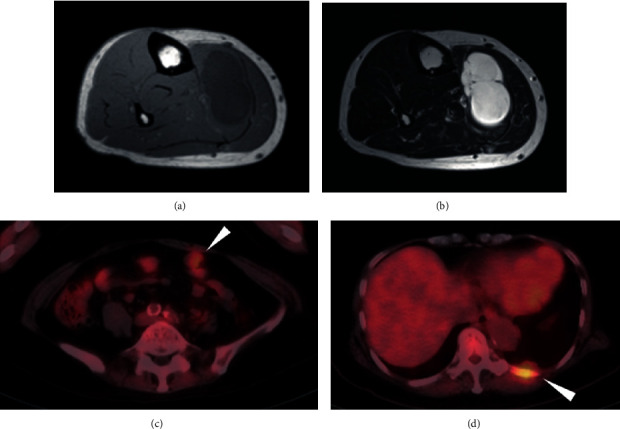
Metastatic tumors in the lower right leg, abdominal space, and chest wall. (a) T1-weighted and (b) T2-weighted MRI showing metastatic tumor in the lower right leg. Metastasis in (c) abdominal space and (d) chest wall in PET.

**Figure 4:**
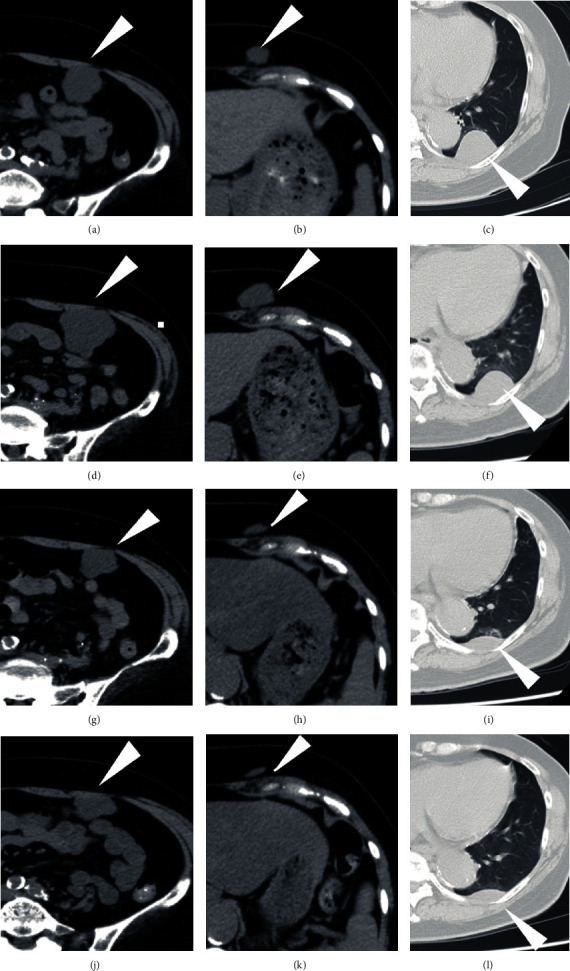
Response to chemotherapy in metastatic myxoid liposarcoma. Computed tomography (a–c) before and (d–f) after 3 cycles of trabectedin showing progressive disease. Partial response was obtained 5 months after treatment with eribulin (g–i). The tumors were under control for another 10 months (j–l).

A 64-year-old female noticed a nodule in her left thigh and visited our institute. A plain radiograph indicated a slight calcification in the posterior middle part of her thigh (Figures 1(a) and 1(b)). Positron emission tomography (PET) revealed a partially moderate uptake inside the tumor (Figure 1(c)). MRI showed a huge, inhomogenous mass (18.7 × 11 × 7.8 cm) associated with a lipomatous lesion (Figures 1(d)–1(f)). From those images, we suspected a malignant lipomatous tumor such as dedifferentiated liposarcoma. We therefore planned to perform a biopsy; however, she refused treatment. Three years later, she revisited us since the tumor had progressively enlarged and skin breakdown had occurred (Figure 2(a)). The tumor had enlarged to 22 × 14 × 9 cm and invaded the neurovascular structures (Figures 2(b) and 2(c)). A needle biopsy revealed myxoid liposarcoma. As a limb-salvage procedure seemed impossible, we performed hip disarticulation (Figure 2(d)). However, she refused adjuvant chemotherapy. Two years postoperation, multiple metastases appeared in the lower right leg, left chest wall, abdominal subcutis, and intra-abdominal space (Figure 3). After surgical resection of the leg tumor, she accepted chemotherapy, so we administered trabectedin as first-line chemotherapy without administrating adriamycin because of her cardiac dysfunction. Three cycles of trabectedin (dose: 1.2 mg/m^2^, 24 hours intravenously from a central venous catheter; no remarkable adverse effect including hematotoxicity was observed) were conducted, resulting in progressive disease (Figures 4(a)–4(f)). We subsequently switched the regimen to eribulin (dose: 1.4 mg/m^2^, intravenously), which evinced a partial response (Figures 4(g)–4(l)). That antitumor effect has lasted now for more than 2 years. So far, no drug resistance has been seen. Adverse effects have been minimal, and the patient has been very satisfied.

## 3. Discussion

In this case report, we reported on the long-lasting, antitumor effect of trabectedin-resistant myxoid liposarcoma, which has been reported to be highly sensitive to trabectedin [9], but the mechanism underlying this resistance has not been fully understood.

Trabectedin is a natural alkaloid derived from the Caribbean tunicate, *Ecteinascidia turbinata.* It has multiple, complex mechanisms of action and, consequently, the potential for extensive clinical application. The first phase III trial to compare trabectedin with dacarbazine in patients having advanced liposarcoma or leiomyosarcoma after prior therapy with an anthracycline and at least one additional systemic regimen showed that the median progression-free survival (PFS) is 4.2 months for trabectedin compared with 1.5 months for dacarbazine, although median overall survival (OS) is 12.4 months for trabectedin vs. 12.9 months for dacarbazine [10].

Eribulin mesylate is a synthetic derivative of halichondrin B, which is isolated from a marine sponge. Its mechanism of action is through microtubule inhibition, which is different from that of taxanes. Eribulin has been approved for the treatment of metastatic breast cancer and more recently for inoperable or metastatic liposarcoma in patients who have received prior anthracycline chemotherapy. The major side effects of eribulin are bone marrow suppression including neutropenia, leukopenia, anemia, and fatigue/weakness, which can be easily managed.

A phase III study comparing eribulin with dacarbazine in patients with advanced liposarcoma or leiomyosarcoma showed a significant improvement in OS for the eribulin arm, with a manageable toxicity profile. Median OS was significantly improved: 13.5 vs. 11.5 months with eribulin vs. dacarbazine, respectively. PFS was not significantly different: 2.6 months for eribulin vs. 2.6 months for dacarbazine [11].

Pazopanib, a multitargeted tyrosine kinase inhibitor, has single-agent activity in patients with advanced nonadipocytic soft tissue sarcoma. Pazopanib for metastatic soft tissue sarcoma (PALETTE): a randomized, double-blind, placebo-controlled phase 3 trial showed that median PFS was 4.6 months for pazopanib compared with 1.6 months for the placebo, although the OS rate was 12.5 months with pazopanib vs. 10.7 months with the placebo [12].

Regarding the evaluation of chemotherapy, in some cases, trabectedin takes very long to be effective and there is even an apparent (paradoxical) increase in the tumor size after the initial cycles that are generally followed by a subsequent shrinkage of the neoplastic lesions. A previous paper mentioned that pseudoprogression may occur in inoperative soft tissue sarcoma in response to trabectedin [13]. They stated that a frequent (*n* = 6/31; 19.3%) pattern of response was tumor liquefaction. However, in our case, progressive disease was apparently observed during trabectedin while tumor shrinkage happened as soon as initiation of eribulin. Hence, tumor shrinkage was caused by eribulin, not trabectedin.

Some reports have mentioned that HMGA1 and UVSSA are involved in resistance to trabectedin [14, 15]. The in vivo patient-derived xenograft model investigating acquired resistance has suggested that the genes involved in lipid processes and adipogenesis had been altered between sensitive and resistant tumors [14]. Further investigation may unveil the detailed mechanism involved in trabectedin-related resistance.

The mechanism of eribulin seems to be unique in various ways. First is the effect on microtubule dynamics to pause microtubule growth and inhibit growth of the microtubules [16]. The second involves angiogenesis and vascular remodeling [17]. In clear-cell sarcoma, tumor reoxygenation caused by eribulin-induced vascular remodeling attenuates cell growth and inhibits ERK1/2 activity to upregulate microphthalmia-associated transcription factor expression and promote melanocytic differentiation [18]. Another is to inhibit epithelial-to-mesenchymal transition. A previous paper has demonstrated that eribulin treatment decreases expression of several mesenchymal marker genes and increases expression of several epithelial markers [19]. The last is a telomerase reverse transcriptase- (TERT-) related mechanism. Eribulin-sensitive cells showed a higher tendency for sphere formation, which is characterized by an enhanced cancer stem cell- (CSC-) like phenotype; those cells expressed a higher level of TERT [20]. The precise mechanism of eribulin remains to be determined.

Eribulin causes fewer complications such as hematotoxicity [21]; hence, it is comparatively safe; it can be used even in the elderly. There are no reports of adverse events in long-term use, but long-term treatment of women with metastatic breast cancer heavily pretreated with eribulin has been reported [22]. Eribulin also may provide long-lasting effects in our case, although we should watch for such long-term complications as secondary tumorigenesis during follow-up.

## 4. Conclusion

We experienced a remarkable response in trabectedin-resistant myxoid liposarcoma. Eribulin should be considered a promising anticancer drug in the treatment of myxoid liposarcoma.

## Data Availability

No data were used to support this study.

## References

[1] Knebel C., Lenze U., Pohlig F. (2017). Prognostic factors and outcome of liposarcoma patients: a retrospective evaluation over 15 years. *BMC Cancer*.

[2] Dalal K. M., Antonescu C. R., Singer S. (2008). Diagnosis and management of lipomatous tumors. *Journal of Surgical Oncology*.

[3] Fletcher C. D. M., Unni K. K., Mertens F. (2002). *Pathology and Genetics of Tumours of Soft Tissue and Bone. WHO Classification of Tumours*.

[4] Nishida Y., Tsukushi S., Nakashima H., Ishiguro N. (2010). Clinicopathologic prognostic factors of pure myxoid liposarcoma of the extremities and trunk wall. *Clinical Orthopaedics and Related Research*.

[5] Chowdhry V., Goldberg S., DeLaney T. F. (2018). Myxoid liposarcoma: treatment outcomes from chemotherapy and radiation therapy. *Sarcoma*.

[6] Judson I., Verweij J., Gelderblom H. (2014). Doxorubicin alone versus intensified doxorubicin plus ifosfamide for first-line treatment of advanced or metastatic soft-tissue sarcoma: a randomised controlled phase 3 trial. *The Lancet Oncology*.

[7] Volkova M., Russell R. (2012). Anthracycline cardiotoxicity: prevalence, pathogenesis and treatment. *Current Cardiology Reviews*.

[8] Demetri G. D., Schöffski P., Grignani G. (2017). Activity of eribulin in patients with advanced liposarcoma demonstrated in a subgroup analysis from a randomized phase III study of eribulin versus dacarbazine. *Journal of Clinical Oncology*.

[9] Jones R. L., Fisher C., Al-Muderis O., Judson I. R. (2005). Differential sensitivity of liposarcoma subtypes to chemotherapy. *European Journal of Cancer*.

[10] Demetri G. D., von Mehren M., Jones R. L. (2016). Efficacy and safety of trabectedin or dacarbazine for metastatic liposarcoma or leiomyosarcoma after failure of conventional chemotherapy: results of a phase III randomized multicenter clinical trial. *Journal of Clinical Oncology*.

[11] Schöffski P., Chawla S., Maki R. G. (2016). Eribulin versus dacarbazine in previously treated patients with advanced liposarcoma or leiomyosarcoma: a randomised, open-label, multicentre, phase 3 trial. *The Lancet*.

[12] van der Graaf W. T. A., Blay J.-Y., Chawla S. P. (2012). Pazopanib for metastatic soft-tissue sarcoma (PALETTE): a randomised, double-blind, placebo-controlled phase 3 trial. *The Lancet*.

[13] Esser M., Kloth C., Thaiss W. M. (2018). CT-response patterns and the role of CT-textural features in inoperable abdominal/retroperitoneal soft tissue sarcomas treated with trabectedin. *European Journal of Radiology*.

[14] Bello E., Brich S., Craparotta I. (2019). Establishment and characterisation of a new patient-derived model of myxoid liposarcoma with acquired resistance to trabectedin. *British Journal of Cancer*.

[15] Loria R., Laquintana V., Bon G. (2018). HMGA1/E2F1 axis and NFkB pathways regulate LPS progression and trabectedin resistance. *Oncogene*.

[16] Jordan M. A., Kamath K., Manna T. (2005). The primary antimitotic mechanism of action of the synthetic halichondrin E7389 is suppression of microtubule growth. *Molecular Cancer Therapeutics*.

[17] Agoulnik S. I., Kawano S., Taylor N. (2014). Eribulin mesylate exerts specific gene expression changes in pericytes and shortens pericyte-driven capillary network in vitro. *Vascular Cell*.

[18] Nakai S., Tamiya H., Imura Y. (2020). Eribulin suppresses clear cell sarcoma growth by inhibiting cell proliferation and inducing melanocytic differentiation both directly and via vascular remodeling. *Molecular Cancer Therapeutics*.

[19] Yoshida T., Ozawa Y., Kimura T. (2014). Eribulin mesilate suppresses experimental metastasis of breast cancer cells by reversing phenotype from epithelial–mesenchymal transition (EMT) to mesenchymal–epithelial transition (MET) states. *British Journal of Cancer*.

[20] Yamaguchi S., Maida Y., Yasukawa M., Kato T., Yoshida M., Masutomi K. (2014). Eribulin mesylate targets human telomerase reverse transcriptase in ovarian cancer cells. *PLoS One*.

[21] Ro J., Cheng F. T. F., Sriuranpong V. (2016). Patient management with eribulin in metastatic breast cancer: a clinical practice guide. *Journal of Breast Cancer*.

[22] Morritti M., Iodice G., Melaccio A. (2017). Long-term treatment with eribulin in heavily pretreated women with metastatic breast cancer: a case series. *Future Oncology*.

